# Sociodemographic Drivers of Accelerated Biological Aging in Mexico City: A Prospective Analysis of 148,110 Adults

**DOI:** 10.1093/geroni/igaf122.165

**Published:** 2025-12-31

**Authors:** Carlos Fermin-Martinez, Omar Yaxmehen Bello-Chavolla, Neftali Eduardo Antonio-Villa, Daniel Ramirez-Garcia, Jeronimo Perezalonso Espinosa

**Affiliations:** Instituto Nacional de Geriatria, Mexico City, Distrito Federal, Mexico; Instituto Nacional de Geriatria, Mexico City, Distrito Federal, Mexico; National Institute of Cardiology Ignacio Chavez, Mexico City, Distrito Federal, Mexico; Instituto Nacional de Geriatria, Mexico City, Distrito Federal, Mexico; Instituto Nacional de Geriatria, Mexico City, Distrito Federal, Mexico

## Abstract

AnthropoAge is a metric designed to estimate biological age using simple anthropometric measures (weight, height, waist circumference), developed and validated in NHANES and in diverse longitudinal aging studies. Here, we sought to characterize AnthropoAge as a predictor of all-cause and cause-specific mortality in the Mexico City Prospective Study (MCPS), and to explore sociodemographic determinants associated with accelerated biological aging, which could influence the aging process in Mexican population. We analyzed 148,110 MCPS participants, including sociodemographic, anthropometric, and health information, with a subset re-evaluated 15 years later. We used c-statistic from Cox models to compare the predictive accuracy for mortality of AnthropoAge and chronological age (CA). Determinants of accelerated aging were assessed with logistic models at baseline, and with mixed-effects linear models after 15 years. AnthropoAge independently increased all-cause mortality risk (HR: 1.059, 95%CI: 1.054-1.064) and showed greater predictive accuracy (c-statistic: 0.752) than CA (0.744), and for major causes of death, including cardiovascular, respiratory, and renal mortality. Accelerated aging was associated at baseline with lower education level (OR: 2.07, 95%CI: 1.98-2.18), higher income (0.82, 0.77-0.86), social development index (0.64, 0.57-0.72), sedentary lifestyle (1.67, 1.62-1.72) and number of comorbidities (1.23, 1.21-1.25). When assessing changes in age acceleration after 15 years, lower education, residence in low-development areas, sedentary lifestyle, and comorbidity burden remain as predictors. Our findings highlight the value of AnthropoAge to characterize age-related outcomes and sociodemographic drivers of the aging process in Mexicans and vulnerable populations. Its application could inform public health strategies and interventions targeting aging-related disparities in middle-income countries.

